# Understanding, Investigating, and promoting deep learning in language education: A survey on chinese college students' deep learning in the online EFL teaching context

**DOI:** 10.3389/fpsyg.2022.955565

**Published:** 2022-09-07

**Authors:** Ruihong Jiang

**Affiliations:** College of Humanities and Arts, Tianjin University of Finance and Economics Pearl River College, Tianjin, China

**Keywords:** deep learning, the motivation of deep learning, the engagement of deep learning, the strategy of deep learning, the directional competence of deep learning, online EFL teaching context, language education

## Abstract

This study aims to (1) develop and validate the four-dimension model hypothesis of deep learning to better understand deep learning in language education; (2) investigate and promote deep learning by conducting a survey involving 533 college students in the online learning English as a foreign language (EFL) teaching context in China. Concretely, this study initially synthesized theoretical insights from deep learning in the education domain and related theories in the second language acquisition and thus proposed the four-dimension model hypothesis of deep learning involving the motivation of deep learning, the engagement of deep learning, the strategy of deep learning, and the directional competence of deep learning. This study subsequently undertook a questionnaire survey utilizing a standardized instrument to confirm the model hypothesis and further investigate the current status and salient differences in students' deep learning in online EFL teaching. Exploratory factor analysis (EFA), confirmation factor analysis (CFA), and Pearson's correlation test validated a positively correlated four-dimension model of deep learning with high composite reliability and good convergent validity. Moreover, the descriptive and inferential statistics revealed that the level of students' deep learning marginally reached the median, with the lowest mean of directional competence and the highest mean of motivation; students manifested more instructional motives, neglect of deploying skilled-based cognitive strategies, and deficiency of language application skills, etc.; there existed some significant differences between deep learning and four sub-dimensions across the grade, English proficiency, EFL course, and vision groups. Eventually, this study proffered primary reasons and five appropriate strategies to scaffold and promote students' deep learning in online EFL teaching. Hopefully, this study will be a pioneering effort to clear away the theoretical muddle of deep learning construct in language education and be illuminating to further improve effectiveness in the online EFL teaching context.

## Introduction

Riding the wave of deep integration of information and communication technology (ICT) and language education, online English as a foreign language (EFL) teaching, which de facto breaks through constraints of time and space between teaching and learning, still suffering from salient difficulties in attaining high teaching quality and ideal learning effect (Panigrahi et al., 2018; Luo et al., 2020). Especially during the COVID-19 pandemic, universities in China have launched sequential semesters of online teaching, proliferating a series of new yet intriguing circumstances worth reflecting on and summarizing: How effectively do students learn in online EFL teaching? How can teachers reduce students' mechanical, surface, and passive learning to scaffold and boost their deep learning in online EFL teaching? Recent trends in the application of online learning through management systems (LMSs), namely Blackboard, have led to a proliferation of studies, ensuring the utility of Blackboard by measuring the perception and use tends to be pivotal to improve the outcome of online EFL learning, especially in the Saudi context (Almekhlafy, 2020; Moawad, 2020). In China, due to the less extensive application of Blackboard in online EFL teaching, studies switch more attention to the urgent need for improving the effectiveness of online EFL teaching by providing supports from teachers or establishing effective learning frameworks (Han et al., 2021; Zhao et al., 2021; Du and Qian, 2022). However, very few studies have been shored up by data-driven analysis of large-scale investigation on the current holistic status of students' language learning in online EFL teaching. As a key trend to boost higher education development in the next 5 years or even longer (Becker et al., 2017), deep learning has become the ultimate target for the integration of ICT and education, as well as a vital dimension to measure learners' learning effect, which is receiving considerable critical attention. Hence, this study attempted to address the aforementioned issues and provide new insights into enhancing teaching quality and learning effect in online EFL teaching through conducting a survey on the current status of deep learning based on an in-depth exploration of the four-dimension model of deep learning and proffering instructional strategies to boost deep learning in the online EFL teaching context.

Deep learning embraces dynamic and versatile theoretical definitions in the education domain. It is conceptualized as a learning method opposite to surface learning (Marton and Säljö, 1976). Subsequently, it is described as a learning process, in which individuals apply learned knowledge in a new context (NRC, 2012). To date, it amounts to an essential competence that students must possess for working and living a civil life in the twenty-first century (Huberman et al., 2014). However, few attempts have been made to clarify the deep learning construct in language education. Although Tochon (2013) has defined a deep approach to language learning as “deep, reflective language learning,” and some researchers have drawn our attention to deep clusters of language learning strategies (Tragant et al., 2013; Zhan et al., 2021); the validity of the theoretical model of deep learning rarely seems to be explored but just to interpret “deep” literally or ignore potentially interwoven correlations between deep learning and Second Language Acquisition (SLA) theories. Similarly, without shrugging off the uncertainty of deep learning construct in language education, scholars in china have explored theoretical teaching modes and frameworks to promote deep learning mostly in high school EFL teaching since 2016 (Luo et al., 2021; Wang et al., 2021). Overall, previous studies have suffered from a lack of clarity in the deep learning construct in language education. On this occasion, few large-scale empirical studies have been performed to investigate students' deep learning with standardized measurement instruments in the EFL teaching context. Consequently, the effectiveness of the modes and frameworks proposed would not be empirically assessed.

The paper begins by reviewing considerable literature on the concept, measurement, investigation, and promotion of deep learning. It will then go on to explicate the four-dimension model hypothesis of deep learning by incorporating correlative SLA theories, considering situated characters of online EFL teaching context. Thereafter, it will present how exploratory factor analysis (EFA) and confirmatory factor analysis (CFA) were exploited in a survey to validate the aforementioned model. It will also summarize the main findings of the additional survey aiming to investigate the current status of deep learning and salient differences in some variables in the online EFL teaching context. The discussion section ties together up all various theoretical and empirical strands to shed light on the four-dimension model of deep learning, survey results, and deep learning promotion strategies.

## Literature review

###  The concept of deep learning

As previous studies suggest, deep learning is originally conceptualized as a learning method. The concept of deep learning is first proposed by Marton and Säljö (1976), which is the opposite of surface learning. It has been noted that students who adopted deep learning methods tend to extract meaning, connect prior knowledge, and think critically. Subsequently, scholars further develop and enrich their theoretical definitions in the education domain: Entwistle and Ramsden (1983) argue that deep learning and comprehension can be displayed in the process of solving and exploring complex problems in unfamiliar situations by combining prior and new knowledge. More attention has also been drawn to the process and essential conditions when explicating deep learning, for example, the learner's identity and connection with the world (Fullan et al., 2019), or learners' motivation and teachers' scaffolding roles (Tochon, 2013). Inspired by “twenty-first-century skills,” the twenty-first century has witnessed the results-oriented research of deep learning, which emphasizes the cultivation of learners' critical thinking and problem-solving ability, creativity and innovation, and communication and cooperation (Asikainen, 2014; Esteban-Guitart and Gee, 2020; Faranda et al., 2021). It is He and Li (2005) who first introduced the concept of deep learning to the education domain in China. Although theoretical research into the deep learning concept from diverse perspectives in the education domain has been deepened, the theoretical muddle of deep learning construct in language education in China has not been cleared away. This study attempted to establish the theoretical model of deep learning in online EFL teaching context and explore the potential theoretical connections between deep learning in the education field and the realm of SLA.

###  The measurement and investigation of deep learning

There has been a considerable amount of research in the measurement and investigation of deep learning by mainly measuring the learning results and process. To illustrate, Biggs (1987) deployed a learning process questionnaire, SPQ, to measure motivation and learning strategies from three different dimensions (i.e., deep approach, surface approach, and achieving approach), which is later revised by removing the achieving approach (Biggs et al., 2001). Different from aforesaid methods and scales, emphasizing the quality and needs of talents cultivation in the twenty-first century, incorporating the interpersonal and individual capacities into measurement, American Institutes for Research(AIR) has built a six-dimension measurement framework to break through the cognitive boundaries of deep learning in the canonical sense (Huberman et al., 2014). In China, some attempts have been made to develop localized scales to measure deep learning more specifically and pertinently in ICT-assisted education in China, albeit a limited amount. For example, Li et al. (2018) creatively incorporate learning engagement into the measurement model to measure students' deep learning in blended teaching at universities. However, in-depth studies on well-established measurement models and standardized scales of deep learning in the realm of language education, especially in the context of online EFL teaching, are relatively scanty. In effect, the Revised Study Process Questionnaire (R-SPQ) (Biggs et al., 2001) has been directly deployed to measure language learners' deep learning (Jiang, 2008), which may suffer from the slightly weak theoretical foundation of SLA and neglect of situated characters of EFL teaching in China, although the R-SPQ is a well-established scale in education *per se*. To address these gaps, this study constructed a deep learning measurement model and drew on a self-developed questionnaire based on the in-depth theoretical exploration of deep learning in language teaching.

Furthermore, to date, a large and growing body of studies has attempted to unravel whether and how some influential factors, such as individual variables (e.g., age, experience, etc.), capacities (e.g., learning, reflection, communication, etc.), self-efficacy, teacher autonomy, or emotional support, etc., influence deep learning (Groves, 2005; Papinczak et al., 2008; Leung et al., 2012; Yang, 2018; Liu et al., 2021; Zhao and Qin, 2021). So far, there have been fewer systematic discussions on deep learning differences across variables in online EFL teaching, for instance, learners' language proficiency, EFL courses, grades, visions, etc., which would be tentatively explicated in this study.

###  Promoting deep learning in language education

Additionally, there is a consensus among many empirical studies that deep learning can be boosted by conducting efficient instructional strategies along with information technology, for example, educational games, creative podcasts, etc. (Vos et al., 2011; Pegrum et al., 2015). Pertaining to language education, with ubiquitous technology application, growing attention is paid to the supporting impacts of technologies on deep language education and learning (Beckett and Iida, 2006; Tochon et al., 2014; Du and Qian, 2022). To illustrate, Tochon et al. (2014) analyzed the impact of online personalized learning on developing deeper levels of language apprenticeship. Likewise, the recent 6 years in China have witnessed a growing amount of theoretical exploration on promoting language learners' deep learning (Luo et al., 2021; Wang et al., 2021). Surprisingly, from the perspective of deep learning strategy, Zhan et al. (2021) conducted an empirical study to elaborate on the interaction between learning motives and self-efficacy in using deep language learning strategies, which is one of few empirical studies aiming to promote learners' deep learning in EFL teaching and is one of little research based on the in-depth and comprehensive theoretical interpretation of SLA theories. Conversely, in the vast majority of aforementioned studies, teaching strategies and modes are proposed theoretically without data-driven analysis and theoretical integration with SLA. Realizing gaps in the extant literature, more multivariate empirical research with sufficient data support and a solid theoretical basis is needed to unravel how to enhance learners' deep learning, especially in the context of online EFL teaching.

###  The four-dimension model hypothesis of deep learning in the online EFL teaching context

By tracing the theoretical development of deep learning in the education domain, deep learning can be considered as a multi-faceted competence that comprises specific learning behaviors and approaches aiming to attain the ultimate goal and can further be extended as a pivotal goal of teaching technology and educational reform, as well as a mainstream orientation of talent cultivation in the twenty-first century. Looking at the complete process of language learning, incorporating diverse definitions of deep learning in previous literature, the present study suggested that deep learning in language education is usually driven by language learning motivation, aims to acquire an in-depth understanding of language knowledge, and attain comprehensive language application skills, complex problem-solving skills in an authentic context, language knowledge transfer and application, critical and higher-order thinking, etc., through performing active involvement and effective learning strategies in the whole process. In this sense, the current study presumed that deep learning in the online EFL teaching context has considerable potential as a multi-dimension model that meaningfully units four main components, namely the motivation of deep learning, the engagement of deep learning, the strategy of deep learning, and the directional competence of deep learning (model hypothesis). Based on the literature on deep learning, incorporating related SLA theories and situated traits of the online EFL teaching, this model would be delineated in a logical and exhaustive manner below. This study also proposed a deep learning measurement model to verify the hypothesis empirically and to further investigate the current status of deep learning, which will be elaborated in the Method section.

#### The motivation of deep learning

As Entwistle (2013) argues, the motivation of deep learning aims to energize and direct learners to pursue the meaning of knowledge. With the support of motivations, learners can consciously establish connections among different knowledge and maintain interest in the learning process, otherwise, it tends to become challenging to access deep learning. It is similarly highlighted that deep learning involves interest and willingness to experience and participate in the learning (Biggs, 1987). Furthermore, the motivation of deep learning can be perceived as intrinsic motivation (Ryan and Deci, 2000b) or a strong sense of identity around goals or passions in deep learning (Fullan et al., 2019). Coincidentally, language learning motivation is considered a vital factor resulting in the sustained process of language learning (Gardner, 1985; Ellis and Ellis, 1994). Therefore, intense learning motivation can become the driving force for deep language learning, which signifies that the motivation of deep learning is one indispensable component. Based on Gardner (1985) motivation theory, the motivation in this study incorporates an integrative motive, which concerns the openness to and respect for targeted cultural groups and ways of life, and gradually develops as the extended or metaphorical or imaginary integration (Dörnyei, 2006). Moreover, considering EFL course curriculum, current talents training needs, societal employment factors, etc., instrumental motive, which is related to concrete benefits that language proficiency might generate (Swann et al., 2010), such as passing a standardized college English test (CET4/CET6), is also taken into account.

#### The engagement of deep learning

Ryan and Deci (2000a) endorse that deep learning is a process of active learning, in which students' active participation and investment are very important (Biggs, 1987). Nevertheless, regarding online EFL teaching, it is usually disturbed by inevitable factors such as long-distance separation, hardware equipment malfunction, network information interference, etc., which can more easily undermine students' long-term and high-quality engagement and result in a high rate of dropping out (Chapelle, 2019). Consequently, it is difficult for students to comprehend knowledge deeply, and even more challenging to further apply, resulting in the failure of attaining deep learning. Hence, the engagement of deep learning is another key component in this model, which mainly concerns behavioral engagement, one aspect of the three-dimension study engagement constructs proposed by Fredricks et al. (2004). It concretely refers to involvement and learning behaviors in academic and social or extracurricular activities aiming to attain positive outcomes (Fredricks et al., 2004). As a whole, online EFL teaching can be seen as a “combo” comprising three stages, namely pre-class, in-class (i.e., online-teaching), and after-class. Thus, pre-class engagement and after-class engagement, for instance, students preview content before online class and revise key questions after class, should not be ignored. The reason why the other two aspects (i.e., emotional engagement and cognitive engagement) are not referred to here is that their definitions, to some extent, overlap with what is portrayed in the motivation dimension and the strategy dimension in this study. As Fredricks et al. (2004) argue, interest and value in emotional engagement overlap considerably with constructs used in motivational literature. Cognitive engagement may suffer from a similar dilemma where the overlap exists in the learning strategy literature.

#### The strategy of deep learning

As Marton and Säljö (1976) argue, learning strategy in deep learning is not mechanical processing in surface learning, but oriented to deep learning, for instance, combining thoughts into a whole structure, critically evaluating the knowledge, reflecting, etc. Similarly, language learning strategy (LLS) is regarded as one of the key factors determining EFL learning (Oxford, 2016) and is considered as “actions chosen by learners (either deliberately or automatically) for the purposes of learning or regulating the learning of language” (Griffiths, 2015). Deep LLS, different from surface LLS, requires the use of high-order skills rather than memorization and repetition (Tragant et al., 2013). Previous studies have demonstrated that deep LLS, namely metacognitive and cognitive strategies, are more applied by successful language learners than memory strategies (Lai, 2009; Gerami and Baighlou, 2011), which to some extent demonstrates the capacity of deep LLS in promoting efficient and deep language learning. However, few studies have further verified which specific type of deep LLS can have a direct predicting role in deep language learning. It is assumed that aiming to access deep learning, learners would tend to exploit more deep LLS. Based on the six-group strategy inventory for language learning (SILL) (Oxford, 1996), which is extensively used to categorize language learning strategies, the strategy of deep learning in the current study mainly refers to deep LLS, including cognitive and metacognitive strategies, social and emotional strategies. Concretely, the cognitive strategies might refer to utilizing in-depth analysis, generalization, induction, deduction, etc., to enhance comprehension of knowledge and promote further application; the metacognitive strategies might underscore regulating individual language learning process through assessing, reflecting, monitoring, etc.; emotional and social strategies might be exploited to manage feelings in language learning or involve in interaction with others aiming to promote mutual learning. It is noteworthy that this study intentionally added surface LLS (e.g., memory strategies) to the follow-up measurement mode of the strategy dimension to be analyzed by EFA and CFA, for better empirically exploring if deep LLS can play an exclusive role in helping learners access deep learning rather than surface LLS.

#### The directional competence of deep learning

Result-oriented deep learning studies pay more attention to attaining the ultimate goals of deep learning, namely cultivating of competencies of deep learning aiming to meet talents requirements of social development (Esteban-Guitart and Gee, 2020), as exemplified by a considerably systematic and compatible six-dimension framework of deep learning competence proposed by AIR (Huberman et al., 2014). Those competencies can also be concretely identified as a broader understanding of knowledge, seeking meaning between content, connecting ideas with prior knowledge and daily experience, collaborating with others, and other various advanced competencies (Asikainen, 2014; Faranda et al., 2021). Therefore, the directional competence acquired by learners through deep learning is the last component. Additionally, pertaining to online language education, a considerable amount of literature has underlined the significance of cementing students' language knowledge, cultivating their 2L proficiency and cross-culture communication, promoting learner autonomy, etc. (Chen and Yang, 2016; Plonsky and Ziegler, 2016; Lai, 2019; Tseng et al., 2020). Hence, in the online EFL teaching context, the directional competence of deep learning can be embodied in a solid foundation of language knowledge and comprehensive language application competence, namely, an in-depth understanding of language knowledge, a capacity to apply language knowledge in specific situations to solve novel practical problems, learning autonomy, English critical thinking, etc.

## Purpose of the study

The main purpose of this study is to develop and validate a four-dimension model of deep learning in the online EFL teaching context with considerable theoretical and empirical evidence to better understand deep learning in language education. The present study also attempts to investigate the current status of college students' deep learning and assess the effects of diverse variables on deep learning, as well as propose appropriate instructional strategies to boost students' deep learning in the online EFL teaching context in China, hopefully shedding new light on promoting teaching quality and learning effect in the realm of ICT-assisted language education. Specifically, the present study attempts to validate one model hypothesis and address five research questions:

- Model Hypothesis: The four-dimension model of deep learning in the online EFL teaching context units four main components, namely the motivation of deep learning, the engagement of deep learning, the learning strategies of deep learning, and the directional competence of deep learning.
- RQ1: What are the dimensions and internal correlations of deep learning in the online EFL teaching context?
- RQ2: What is the overall current status of college students' deep learning in the online EFL teaching context in China?
- RQ3: Are there statistically significant differences in deep learning or other sub-dimensions across the grade, English proficiency, EFL course, and vision groups?
- RQ4: What might be the main reasons behind survey results?
- RQ5: What are efficient strategies to boost students' deep learning in the online EFL teaching context?

To achieve above the aims, this study principally conducted a survey by a self-developed and standardized questionnaire method and also drew on EFA, CFA, descriptive analysis, independent sample *t*-test, and ANOVA to analyze the quantitative data, which would contribute to deeper insights into deep learning in the online EFL teaching context.

## Materials and methods

###  Participants

Participants of this questionnaire were college students attending online EFL courses this spring semester at one university in Tianjin, China. Since EFL courses are exclusively compulsory for freshmen and sophomores according to national initiatives in China, the stratified random sampling was just conducted in the above two grades. In total, 533 students in two grades (52.1% freshmen and 47.9% sophomores), 4 institutes, and 10 majors were selected as samples. The sample was 72.3% women and 27.7% men with ages ranging from 17 to 20. The sample distributions were relatively balanced. Concretely, participants were attending three types of EFL courses: 81.8% public college English course (PCE); 0.09% ELS course targeting students in the international program; and 0.09% basic English course targeting students majoring in teaching Chinese as a foreign language (TCFL). Participants also reported their English proficiency as 71.5% primary level (non-passing CET-4 test); 26.1% intermediate level (passing CET-4 test); and 0.02% intermediate and advanced level (passing CET-6 test). Three sorts of visions after graduation were presented: hunting for a job (49.58%); further studying for a master's degree in the domestic or overseas (45.99%); and self-employment (4.43%). In total, 533 questionnaires were issued online and 474 valid questionnaires were retrieved, with the effective response rate being approximately 88.93%. Therefore, this data analysis results were representative.

###  Instrument

#### College students' deep learning in online EFL teaching questionnaire

The questionnaire comprises two main parts: basic information questions (i.e., gender, grade, English proficiency, EFL course, and vision) and closed-ended questions on deep learning, of which responses are provided using a 5-point Likert scale ranging from 1 (Strongly Disagree) to 5 (Strongly Agree).

Posited on model hypothesis, the deep learning measurement model involves four sub-dimensions, namely the motivation, the engagement, the strategy, and the directional competence. The second part of this questionnaire was developed based on the above measurement model. Some related scales with good reliability and validity were referred to when compiling items. The items were translated into Chinese for higher readability and comprehension and were adjusted and contextualized for authentic online EFL teaching contexts. More details are demonstrated below.

The motivation measurement sub-dimension aims to measure learners' language learning motivation directed to deep learning, which mainly involves integrative motive and instrumental motive (Gardner, 1985), based on definitions and classification of 2L motivation in SLA. Referring to motivation questionnaire (Dörnyei and Taguchi, 2009), considering situated characters of the online EFL teaching in China, items were revised and contextualized. The sample item of integrative motives is “I think it's important to learn English to know more about the culture and arts of its speakers.” The sample item of instrumental motives is “I study English diligently to pass standardized English tests (e.g., CET4, CET6, TOEFL, IELTS, etc.).”

The engagement measurement sub-dimension aims to measure participants' behavioral engagement. Items were adapted from the National Survey of Student Engagement 2020, which is a well-confirmed and widely used study engagement questionnaire for college students and can also be applied in online teaching (Robinson and Hullinger, 2008). Engagement in three stages of online EFL teaching was portrayed in specific items. The sample item is “After online class, I review and summarize key ideas or concepts.”

The strategy measurement sub-dimension aims at measuring cognitive and metacognitive strategies, along with social and emotional strategies according to deep LLS research in SLA (Tragant et al., 2013). Items were adapted from SILL, some of which were simplified and adjusted for better understanding. A sample item of metacognitive is “I regularly reflect on my English learning to avoid making similar mistakes.” A sample item of cognitive strategy is “I look for words in my own language that are similar to new words in English.” A sample item of social strategy is “I participate in group discussions with students to better understand learning content from different perspectives.” A sample item of emotional strategy is “I watch inspiring online English videos (e.g., speech, motive, social media video, etc.) to encourage myself in English learning.” Aiming to test the exclusively crucial effect of deep LLS in promoting deep learning, some surface LLS items were added to the questionnaire for further discussion on the results of CFA and EFA. A sample item of memory strategy is “I use online English learning apps to remember new words.”

The directional competence measurement sub-dimension aims to measure various advanced competences acquired by learners. Based on six dimensions of deep learning competence proposed by AIR (Huberman et al., 2014), combined with the main teaching objectives of online EFL teaching, items were compiled as the following samples: “I think I have mastery of basic English language knowledge (i.e., vocabulary, grammar, etc.),” “I think I can solve practical problems in English in a specific context.”

### Procedure

After demonstrating the research goals and procedures and asking for permission at the university, a pilot test of the questionnaire with 50 participants was conducted to note any points of confusion. An adapted version was afterward filled out online by 533 participants anonymously, honestly, and voluntarily. None posed any questions or confusion on this questionnaire during the whole process, which indicated that it was properly organized and easy for them to use.

###  Data analysis

A total of 474 pieces of valid data were collected online and then quantitatively analyzed on SPSS.25. and AMOS.24. Specifically, EFA and CFA were initially conducted to test the reliability and validity of the questionnaire and measurement mode, to further verify the model hypothesis. Afterward, Pearson's correlation test was deployed to unravel the internal correlation between four sub-dimensions. Descriptive analysis was conducted to investigate the current status of deep learning in EFL online teaching. Additionally, Independent sample *t*-test and ANOVA were used to explore whether these variables (i.e., grade, English proficiency, EFL course, and vision) can cause statistically significant differences in deep learning.

## Results

###  Exploratory factor analysis

**Figure 1:**
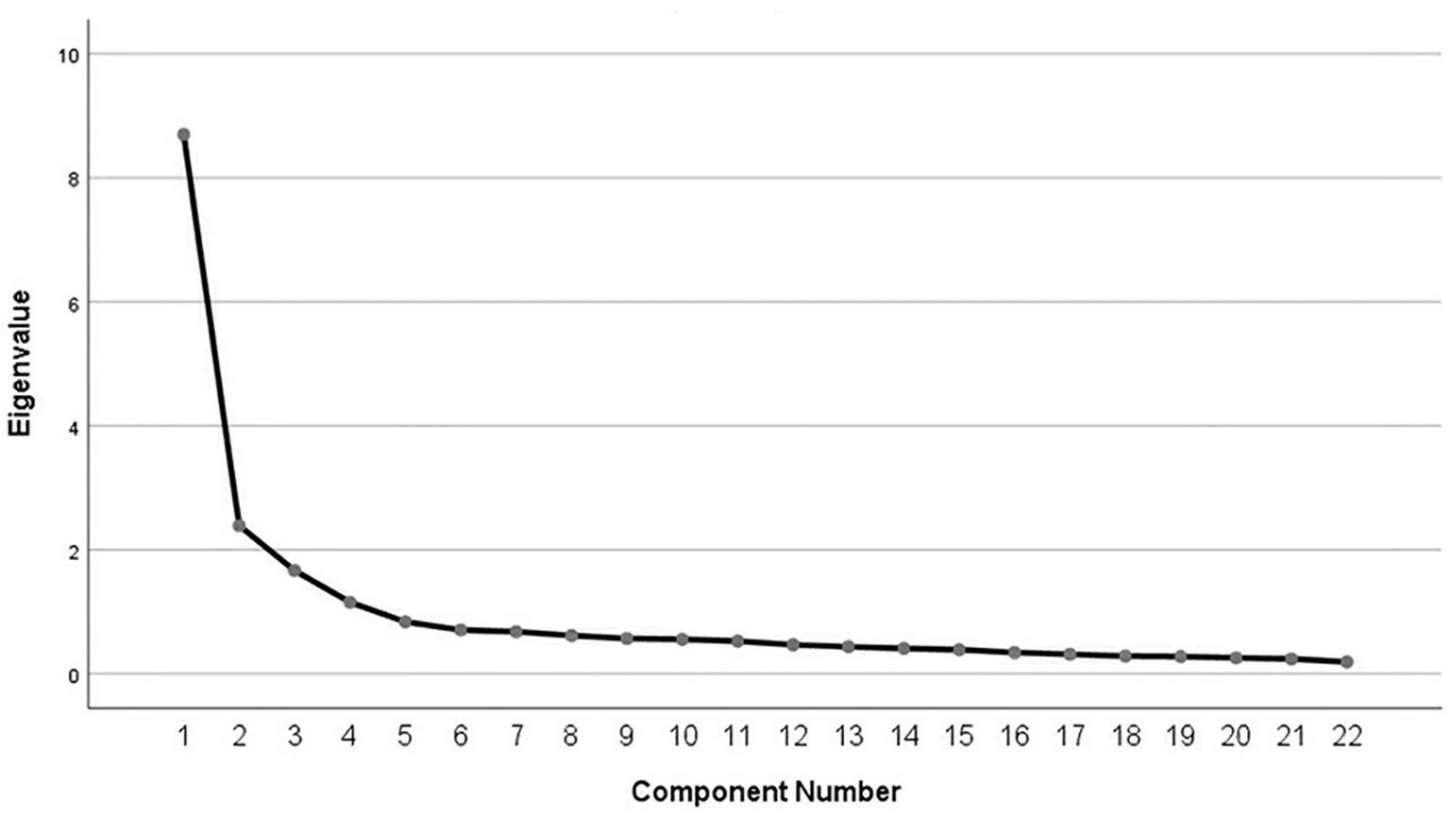
Screen plot.

Table 1 shows that the KMO value was 0.922(>0.50), and Bartlett's spherical chi-square value was 5647.469 (*p* = 0.000 < 0.05), indicating that the questionnaire's factor structure was suitable for EFA (Tabachnick and Fidell, 2001). Through principal component analysis and Varimax with Kaiser normalization rotation method, according to the principle that the eigenvalue is greater than 1, as Table 3 presents, 4 factors with 22 variables were extracted sequentially by deleting variables with less salient loadings (<0.40), cross-loading variables, and factors with less than two variables with less related content to the questionnaire (Dörnyei, 2007). It also corresponded to the four-factor solution in the scree plot in Figure 1, which demonstrates that a useful model for these data may have 4 factors. The standard factor loadings of variables ranged from 0.551 to 0.844 and the total interpretation rate was 63.207% (>50%) in Tables 2, 3.

**Table 1:** KMO and Bartlett's test.

The Kaiser-Meyer-OlKin measurement of sample adequacy		0.922
Bartlett's test of sphericity	Approx. Chi-Square	5647.469
	df	231
	Sig.	0.000

**Table 2:** Total variance explained.

**Component**	**Initial eigenvalues**			**Extraction sums of squared loadings**			**Rotation sums of squared loadings**		
**t**	**Total**	**% of variance**	**Cumulative %**	**Total**	**% of variance**	**Cumulative %**	**Total**	**% of variance**	**Cumulative %**
1	8.697	39.532	39.532	8.697	39.532	39.532	4.173	18.968	18.968
2	2.390	10.862	50.394	2.390	10.862	50.394	3.576	16.254	35.222
3	1.665	7.568	57.962	1.665	7.568	57.962	3.282	14.919	50.141
4	1.154	5.245	63.207	1.154	5.245	63.207	2.874	13.066	63.207
5	0.837	3.806	67.013						

Extraction method: Principal component analysis.

**Table 3:** Rotated component matrix^*a*^.

**Variables**	**1**	**2**	**3**	**4**
C2	0.844			
C4	0.801			
C5	0.789			
C3	0.772			
C6	0.765			
C1	0.717			
S11		0.727		
S3		0.698		
S12		0.687		
S10		0.664		
S2		0.648		
S9		0.619		
E5			0.806	
E6			0.779	
E4			0.764	
E7			0.704	
E2			0.551	
M2				0.728
M3				0.719
M5				0.698
M1				0.657
M4				0.652

Extraction method: Principal component analysis. Rotation method: Varimax with Kaiser normalization.

* Rotation converged in five iterations.

Moreover, as Table 4 shows, Cronbach's alpha coefficients of each factor ranged from 0.799 to 0.907 (>0.70) and Cronbach's alpha coefficient of the overall questionnaire was 0.926 (>0.70), indicating that each sub-dimension and whole questionnaire had high re liability and internal consistency.

**Table 4:** Cronbach's alpha coefficients for four sub-dimensions and the overall scale in exploratory factor analysis (EFA).

	**Factor**	**Alpha coefficients**	**N of Items**
1	Competence	0.907	6
2	Strategy	0.868	6
3	Engagement	0.858	5
4	Motivation	0.799	5
	Deep learning	0.926	22

###  Confirmatory factor analysis

**Figure 2:**
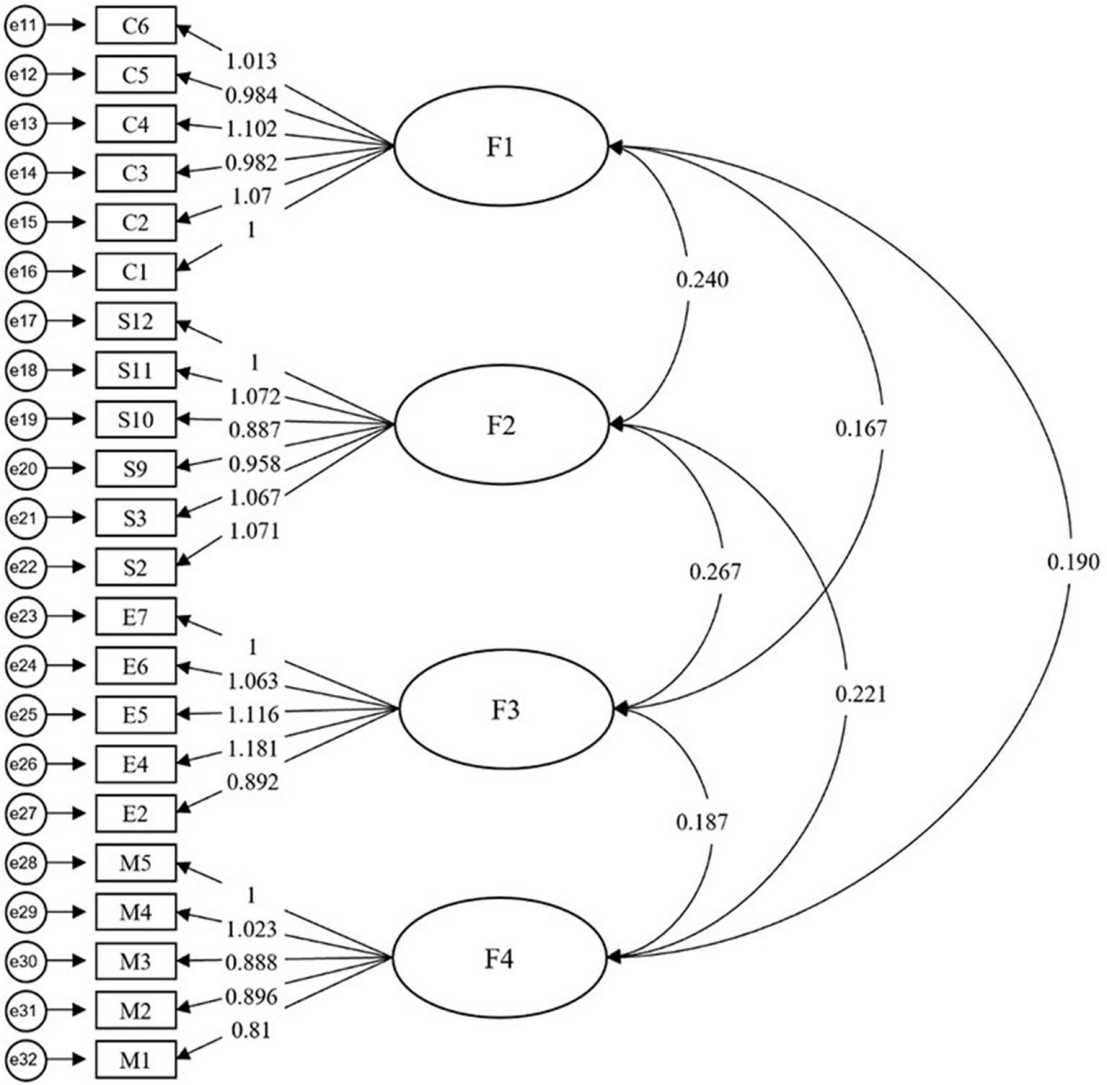
Confirmatory factor analysis (CFA): F1, competence; F2, strategy; F3, engagement; and F4, motivation.

Confirmatory factor analysis was conducted on AMOS.24. to confirm hypothesized factor structure, namely the four-dimension model of deep learning(H1). The model fit statistics indicate a good model fit: χ^2^ = 643.236 (*p* = 0.002); IFI = 0.921 (>0.9); TLI = 0.909 (>0.9); CFI = 0.920 (>0.9); PCFI = 0.809 (>0.5), PNFI=0.780 (>0.5); CMIN/DF = 3.169 (3 <NC <5) and RMSEA = 0.068 (<0.08) (MacCallum et al., 1996). Table 5 and Figure 2 report that the Cronbach's alpha coefficients of each factor were greater than 0.7, corresponding to above Cronbach's alpha reliability test results in EFA, reconfirming the high reliability of the scale. Moreover, the standard loadings of 22 variables in four factors ranged from 0.599 to 0.834 in CFA, similar to the results in EFA. Table 5 and Figure 2 also present that the Composite Reliability values (CR) of each factor were all greater than 0.7, indicating that the model had good composite reliability, and the average variance extracted values (AVE) of each variable were greater than 0.5, except for the F4 (AVE = 0.445), which was still in the acceptable range (0.36–0.50), signifying that the model had good convergent validity.

**Table 5:** Confirmatory factor analysis (CFA).

			**Estimate**	**Std. estimate**	**S.E**.	**C.R**.	**P**	**Cronbach alpha**	**AVE**	**CR**
C6	←	F1	1.013	0.758	0.060	16.900	***			
C5	←	F1	0.984	0.775	0.057	17.330	***	0.907	0.621	0.907
C4	←	F1	1.102	0.812	0.060	18.291	***			
C3	←	F1	0.982	0.785	0.056	17.598	***			
C2	←	F1	1.070	0.834	0.057	18.840	***			
C1	←	F1	1.000	0.760						
S12	←	F2	1.000	0.719						
S11	←	F2	1.072	0.793	0.066	16.350	***			
S10	←	F2	0.887	0.608	0.071	12.554	***			
S9	←	F2	0.958	0.710	0.065	14.666	***	0.868	0.531	0.871
S3	←	F2	1.067	0.760	0.068	15.703	***			
S2	←	F2	1.071	0.766	0.068	15.817	***			
E7	←	F3	1.000	0.714						
E6	←	F3	1.063	0.716	0.073	14.565	***			
E5	←	F3	1.116	0.814	0.068	16.427	***	0.858	0.558	0.862
E4	←	F3	1.181	0.840	0.070	16.877	***			
E2	←	F3	0.892	0.632	0.069	12.882	***			
M5	←	F4	1.000	0.731					
M4	←	F4	1.023	0.729	0.073	13.976	***			
M3	←	F4	0.888	0.599	0.076	11.684	***	0.799	0.445	0.799
M2	←	F4	0.896	0.646	0.071	12.548	***			
M1	←	F4	0.810	0.619	0.067	12.053	***			

F1, competence; F2, strategy; F3, engagement; F4, motivation. ***p < 0.001.

###  Pearson's correlation test

Aiming to explore internal correlations in four factors of deep learning and evaluate the strength and direction of association with each other, Pearson's correlation test was conducted. As Table 6 suggests, four factors displayed a positive pairwise correlation: engagement had a positive correlation with motivation (*R* = 0.457, *p* < 0.01) and competence had a positive correlation with strategy (*r* = 0.547, *p* < 0.01). Engagement was positively related to competence (*r* = 0.388, *p* < 0.01) and strategy (*r* = 0.656, *p* < 0.01). Motivation was positively related to competence (*r* = 0.434, *p* < 0.01) and strategy (*r* = 0.528, *p* < 0.01). Additionally, four factors all displayed a positive correlation with deep learning, respectively (*r* > 0.70, *p* < 0.01). It is the strategy that demonstrated the highest positive correlation with deep learning (*r* = 0. 870, *p* < 0.01).

**Table 6:** Descriptive statistics and correlation among variables.

**Variables**	**N**	**Mean**	**SD**	**M**	**E**	**S**	**C**	**Deep learning**
M: motivation	474	3.9241	0.59438	1				
E: engagement	474	3.1046	0.68929	0.457^**^	1			
S: strategy	474	3.2222	0.64887	0.528^**^	0.656^**^	1		
C: competence	474	2.9170	0.70221	0.434^**^	0.388^**^	0.547^**^	1	
Deep learning	474	3.2718	0.52395	0.731^**^	0.780^**^	0.870^**^	0.778^**^	1

** p < 0.01.

### Descriptive analysis

Table 6 suggests that the mean of deep learning, which equals the average of scores on overall 22 variables in scale, marginally reached the median value (*M* = 3.272, SD = 0.52395). The mean of motivation (*M* = 3.9241, SD = 0.59438) was higher than other sub-dimensions, with the highest score (*M* = 4.2743, SD = 0.85075) in instructional motive (M3) and the lowest score (*M* = 3.5865, SD = 0.75125) in integrative motive (M1) (see Appendix). In contrast, the mean of competence (*M* = 2.9170, SD = 0.70221) was the lowest, even lower than median value, with the highest score (*M* = 3.1709, SD = 0.81627) in learning autonomy(C3) and lower scores (*M* < 0.3) in basic language knowledge, language application skills, problem-solving skills, critically thinking, etc. (C1, C2, C6, C4,) (see Appendix). In strategy dimension, the score in skilled-based cognitive strategies (S10) is the lowest (*M* = 2.8165, SD = 0.87608). Additionally, students performed lower levels in some behavioral engagement (E6, E7), involving interaction and discussion with teachers and peers in or after online class (see Appendix).

### Comparative analysis

An independent sample *t*-test was conducted to compare scores of two grade groups in deep learning and four sub-dimensions. In Leven's test, except for motivation (*p* < 0.05), the study referred to results of assume equal variance in engagement, strategy, competence, and deep learning (*P* < 0.05). Table 7 suggests the scores of motivation (*P* = 0.013), engagement (*P* = 0.024), strategy (*P* = 0.004), and deep learning (*P* = 0.006) of freshmen were statistically higher than those of sophomores. There was no significant difference in scores of competence, which were relatively low for the two groups.

**Table 7:** Independent samples *t*-test of students' deep learning across grade groups.

**Variables**	**Grade**	**N**	**M**	**S D**	**T**	**df**	**Sig(2-tailed)**
Motivation	F	247	3.9895	0.53834	2.495	442.144	0.013
	S	227	3.8529	0.64357			
Engagement	F	247	3.1733	0.66119	2.271	472	0.024
	S	227	3.0300	0.71258			
Strategy	F	247	3.3036	0.57893	2.871	472	0.004
	S	227	3.1336	0.70798			
Competence	F	247	2.9548	0.69428	1.222	472	0.222
	S	227	2.8759	0.70998			
Overall	F	247	3.3347	0.48271	2.748	472	0.006
	S	227	3.2032	0.55846			

F, freshmen; S, sophomores.

ANOVA was conducted to compare scores across three English proficiency groups in deep learning and four sub-dimensions. Leven's test shows except for deep learning (*p* < 0.05), the LSD method can be used for multiple comparisons in others dimensions. In Table 8, ANOVA suggests that in motivation (*P* = 0.002), strategy (*p* = 0.030), competence (*P* = 0.000), and deep learning (*p* = 0.000), there were at least one significant difference amongst the group means. The *post-hoc* test reveals significant differences between PL and IL groups in motivation (*P* = 0.006), strategy (*P* = 0.010), competence (*P* = 0.000), and deep learning (*P* = 0.000): the scores of the IL group were significantly higher than the PL group counterparts. Moreover, the scores of IAL group in motivation (*P* = 0.015), competence (*p* = 0.011), and deep learning (*p* = 0.000) were significantly higher than IP group counterparts. There were no significant differences between the three groups in engagement.

**Table 8:** ANOVA for comparison of students' deep learning across English proficiency groups.

	**M(SD)**				
**Variables**	**PL (*N* = 339)**	**IL (*N* = 124)**	**IAL (*N* = 11)**	***F***	**LSD/Tamhane**
Motivation	3.8690 (0.59551)	4.0403 (0.58434)	4.3091 (0.30151)	6.27^*^	PL < IL (*P* = 0.006), PL < IAL (*P* = 0.015)
Engagement	3.0791 (0.67789)	3.1645 (0.72181)	3.2182 (0.67204)	0.85	
Strategy	3.1726 (0.65294)	3.3481 (0.63616)	3.3333 (0.48305)	3.525^*^	PL < IL(*P* = 0.010)
Competence	2.7915 (0.69655)	3.2245 (0.62903)	3.3182 (0.41803)	20.68^*^	PL < IL (*P* = 0.000), PL < IAL (*p* = 0.011)
Deep learning	3.2057 (0.52088)	3.4300 (0.51427)	3.5248 (0.18109)	9.999^*^	PL < IL (*P* = 0.000), PL < IAL (*p* = 0.000)

* p < 0.05.

PL, primary level group (non-passing CET-4 test); IL, intermediate level group (passing CET-4 test); IAL, intermediate and advanced level group (passing CET-6 test).

In order to examine whether three EFL course groups differ in deep learning and four sub-dimensions, ANOVA was conducted. Leven's test does not show any significant differences in all dimensions (*P* < 0.05), so the LSD method can be used for multiple comparisons. In Table 9, ANOVA suggests that in motivation (*P* = 0.018), engagement (*p* = 0.000), strategy (*P* = 0.010), and deep learning (*P* = 0.005), there was at least one significant difference among the group means. The *post-hoc* test reveals significant differences between PCE and BE groups for motivation (*P* = 0.005), engagement (*p* = 0.000), strategy (*P* = 0.006), and deep learning (*P* = 0.009): the score of BE group was significantly higher than the PCE group counterparts. Besides, the ELS group had higher scores in engagement (*P* = 0.026) than the PCE group, but lower scores in motivation (*P* = 0.045) compared with the BE group. There were no significant differences among the three group in competence.

**Table 9:** ANOVA for comparison of students' deep learning across English as a foreign language (EFL) course groups.

	**M(SD)**				
**Variables**	**PCE (*N* = 388)**	**ELS (*N* = 43)**	**BE (*N* = 43)**	***F***	**LSD**
Motivation	3.8985(0.59498)	3.9116 (0.66376)	4.1674 (0.45759)	4.025^*^	PCE < BE(*P* = 0.005), ELS < BE (*P* = 0.045)
Engagement	3.0412 (0.68044)	3.2837 (0.66222)	3.4977 (0.64641)	10.488^*^	PCE < BE (*p* = 0.000), PCE < ELS (*P* = 0.026)
Strategy	3.1813 (0.64872)	3.3488 (0.64428)	3.4651 (0.59713)	4.676^*^	PCE < BE (*p* = 0.006)
Competence	2.9003 (0.69499)	3.0853 (0.72134)	2.8992 (0.74192)	1.360	
Deep learning	3.2358 (0.52232)	3.3901 (0.52601)	3.4778 (0.48108)	5.433^*^	PCE < BE (*P* = 0.009)

* p < 0.05.

PCE, Public College English course group; ELS, ELS course group; BE, Basic English course group.

At last, ANOVA was used to explore differences in deep learning and four sub-dimensions among three vision groups. Leven's test shows a significant difference in motivation (*P* < 0.05), Tamhane method was conducted to multiply and compare three groups in this dimension, whereas others resorted to the LSD method. In Table 10, ANOVA shows that three group differed significantly in motivation (*P* = 0.000), strategy (*P* = 0.048), competence (*P* = 0.001), and deep learning (*P* = 0.001). As the LSD and Tamhane demonstrate, the scores of the HJ group in motivation (*P* = 0.000), strategy (*P* = 0.030), competence (*P* = 0.000), and deep learning (*P* = 0.000) were, respectively, much lower than the FS group counterparts. FS group also performed better than the SE group in motivation (*P* = 0.000) and competence (*P* = 0.044). There were no statistically significant differences between the HJ group and SE group in the above dimensions. In addition, the three groups did not differ from each other significantly in engagement.

**Table 10:** ANOVA for comparison of students' deep learning across vision groups.

	**M(SD)**				
**Variables**	**HJ (*N* = 235)**	**FS (*N* = 218)**	**SE (*N* = 21)**	***F***	**LSD/Tamhane**
Motivation	3.7932 (0.62590)	4.0862 (0.53512)	3.7048 (0.35563)	16.221^*^	HJ < FS (*P* = 0.000), SE < FS (*P* = 0.000)
Engagement	3.0791 (0.65636)	3.1367 (0.73606)	3.0571 (0.54458)	0.445	
Strategy	3.1610 (0.64455)	3.2936 (0.66355)	3.1667 (0.46547)	2.456^*^	HJ < FS (*P* = 0.030)
Competence	2.8106 (0.70405)	3.0497 (0.67503)	2.7302 (0.74624)	7.534^*^	HJ < FS (*P* = 0.000), SE < FS (*P* = 0.044)
Deep learning	3.1905 (0.52198)	3.3716 (0.52095)	3.1450 (0.39642)	7.600^*^	HJ < FS (*P* = 0.000)

* p < 0.05.

HJ, hunting for a job group; FS, further studying group; SE, self-employment group.

## Discussion

###  The four-dimension model of deep learning in the online EFL teaching context

Utilizing EFA and CFA, this study first empirically validated the four-dimension model hypothesis of deep learning involving the motivation of deep learning, the engagement of deep learning, the strategy of deep learning, and the directional competence of deep learning in the context of online EFL teaching. In particular, engagement, motivation, and strategy were included in this deep learning model, which was congruent with the general definitions of deep learning from cognitive perspectives in the education domain (Marton and Säljö, 1976; Biggs, 1987). The directional competence further underpinned the “results-oriented” concept of deep learning, which is defined as an essential competence for students when working and living a civil life in the twenty-first century (Huberman et al., 2014). Overall, the four-dimension model of deep learning in online EFL teaching well echoed the dominant theoretical conceptualizations of deep learning from both cognitive and talent training needs perspectives in the literature. It is also noteworthy that some emotional factors (e.g., the sense of interest) were implicitly subsumed under the motivation dimension, which, to some extent, shored up the deep learning model including cognitive emotional experiences proposed by Liu et al. (2021). Additionally, Pearson's correlation test demonstrates that these four dimensions interacted with each other in a positively correlated way. Surprisingly, among these four internally correlated dimensions, the strategy had a relatively stronger positive correlation with deep learning than others. To some extent, this suggests that language learning strategies, especially deep LLS, exerted an important role in L2 attainment, but its mechanism in the complex process of learning deserves further exploration (Dörnyei, 2006). Table 11 presents definitions and components of the four dimensions of the deep learning model and more details will be explicated, respectively, below.

**Table 11:** The four dimensions of the deep learning model in the online EFL teaching context.

**Dimensions**	**Definitions**	**Components**
The motivation of deep learning	Learners' strong interests, subjective willingness to learn, and a strong sense of identity around goals or passions, integrative or instrumental, directed to deep language learning	Integrative motives and instrumental motives
The strategy of deep learning	Deep language learning strategies deployed by learners to access deep language cognitive process	Cognitive strategies, metacognitive strategies, and social strategies
The engagement of deep learning	Learners' concrete involvement and learning behaviors aiming to attain positive academic outcomes and avoid alienation at three stages in online EFL teaching	Pre-class engagement, in-class engagement, and after-class engagement
The directional competence of deep learning	The ultimate advanced language competences nurtured in deep language learning	In-depth mastery of language knowledge, language application skills, English critical thinking, problem-solving capacity, learning autonomy, and online English information processing capacity

To start with, the motivation of deep learning was one component of the model, referring to learners' strong interests, subjective willingness to learn, and a strong sense of identity around goals or passions, integrative or instrumental, directed to deep language learning, which further supported general definitions of deep learning (Biggs, 1987; Ryan and Deci, 2000b; Fullan et al., 2019; Esteban-Guitart and Gee, 2020), and highlighted the directing and energizing role of motivation in the long-term process of language learning (Gardner, 1985; Dörnyei, 2006; Tochon, 2013). Results also reveal that the motivation of deep learning comprised integrative motive and instrumental motive, which was in accordance with the long-lived conceptualization of L2 motivation from psychological perspectives (Gardner, 1985; Swann et al., 2010). The rather intriguing finding in the descriptive analysis might be that students may be driven by more instructional motives than integrative motives in deep language learning, for instance, passing English tests, improving overall competitiveness, etc., which was generally in line with those of previous studies (Chen et al., 2005; Liu, 2007; Zhan et al., 2021). To some extent, this finding also mirrors that instructional motives will act out a more promoting role in instructional learning situations especially without any direct contact with native speakers, while “integrative metaphor simply did not make sense” (Swann et al., 2010). Nevertheless, regarding recent 2L motivation studies from a complex dynamic perspective, the current study may be limited to exploring dynamic characters and temporal variation of motivation, which is worthy of further discussion.

The strategy of deep learning was another critical component, referring to deep language learning strategies deployed by learners to access deep language cognitive process, which was opposite to surface learning strategies underscoring mechanical processing (Marton and Säljö, 1976). Inspired by the deep LLS concept concerning high-order skills strategies (Tragant et al., 2013), referring to the six-group SILL (Oxford, 1996), this study assumed that cognitive and metacognitive strategies, along with social and emotional strategies, constituted the strategy of deep learning. Since few works of literature empirically suggest deep LLS can exclusively promote deep learning in the online EFL teaching context, memory strategies were intentionally included in the initial measurement model to verify the above assumption. As expected, EFA reveals that items related to memory strategies (S1, S4, S6, and S7) were not retained due to less salient loadings (< 0.4) on each factor or cross-loading variables, which empirically suggests that surface LLS focusing on memorization and repetition may not contribute to deep language learning. Nevertheless, the most striking finding in EFA was that one factor with just two items about emotional strategies (S8 and S5) was excluded for a much higher total interpretation rate since this factor did not seem to fit the conceptually interpretable four-factor solution. This unexpected finding indicates that from the perspective of learners, strategies to manage feelings in language learning may not play an essential role in attaining deep learning in the context of online EFL teaching. One possible explanation for this might be that compared with emotional strategies deployed by learners on their own, perceived teacher emotional support might perform efficiently in promoting students' deep learning in practical teaching contexts (Karagiannopoulou and Entwistle, 2019; Liu et al., 2021). Therefore, the strategy of deep learning in this study involved cognitive and metacognitive strategies, as well as social strategies, which can be considered as deep LLS contributing to deep learning in the online EFL teaching context.

Additionally, the engagement of deep learning was the third component, referring to learners' concrete involvement and learning behaviors aiming to attain positive academic outcomes and avoid alienation across three stages in online EFL teaching, namely pre-class engagement, in-class engagement, and after-class engagement. This confirmed that deep learning is a process of active learning, in which students' active participation and investment are very critical (Biggs, 1987; Ryan and Deci, 2000a). Concretely, pre-class engagement referred to learning behaviors, such as previewing content and maintaining an active mood before online class. In-class engagement included participation in online academic activities and active interaction with teachers and peers. After-class engagement covered learning behaviors, such as revising and summarizing key knowledge, etc. In addition, the directional competence of deep learning was the last component of the model, referring to the ultimate advanced language competencies nurtured in the deep language learning. In online EFL teaching, these competencies involved in-depth mastery of language knowledge, a capacity to apply language knowledge in specific situations to solve novel problems, learning autonomy, English critical thinking, processing online English information, etc., which are generally agreed with deep learning conceptualization in the literature on result-oriented deep learning (Asikainen, 2014; Huberman et al., 2014; Faranda et al., 2021).

###  The current status of college students' deep learning in online EFL teaching context

Descriptive analysis demonstrates that the current level of students' deep learning in online EFL teaching was marginally median, of which the mean was slightly lower than that in previous surveys in traditional face-to-face general teaching (Yang, 2018). Thus, it is still challenging for college students to access deep learning in the online EFL teaching context, especially during the COVID-19 pandemic. Moreover, results also demonstrate that even though students had strong motivation, they still lacked advanced language competencies, especially language application skills and problem-solving skills. One reason for this might be ascribed to the underestimated self-evaluation of academic success, which was explained as the influence of Chinese modesty (Wan and Lee, 2017; Zhan et al., 2021). But if we offer a glimpse into the strategy dimension, we found that students tended to neglect to deploy skill-based cognitive strategy (S10) during deep learning, thereby hindering the development of language application skills and problem-solving skills. In addition, students presented relatively low engagement regardless of distinct English proficiency and visions, and similarly low competence across grade and EFL course groups, which will be elaborated in the following comparative analysis section. Overall, it is urgent for EFL teachers to seek possible antidotes to the above problems.

###  Comparative analysis

Regarding grade groups, the striking finding was that freshmen performed visibly better than sophomores in motivation, engagement, strategy, and overall deep learning. A possible explanation for this might be that freshmen who just attended National College Entrance Examination (NCEE) and completed an arduous senior year in high school, possibly maintained such intense learning momentum and routines resulting in stronger motivation to learn, higher investment, and more active participation in learning, as well as the higher level of deep learning. In contrast, sophomores may mostly pay more attention to core course learning than EFL learning. Moreover, there existed a significant difference in strategy dimension across grade groups, which was generally in agreement with Tragant et al. (2013), who found that language learning strategies used were significantly different for the two age groups. Concretely, freshmen tended to make more attempts to employ strategies to learn deeply possibly due to intense 3-year instructed language learning in high school aiming to attain high scores in NCEE. However, there was no significant difference in competence, indicating that it might be challenging for both grades to attain advanced language competence in the online EFL teaching context.

In terms of English proficiency groups, results revealed that the higher level of English proficiency students had the higher levels of motivation, strategy, competence, and deep learning. The finding was partly consistent with some research discovering that more proficient EFL learners used deep language learning strategies more frequently than less proficient counterparts (Lai, 2009; Gerami and Baighlou, 2011; Zhan et al., 2021). In this vein, due to the internal positive correlation of four dimensions, more proficient EFL learners seemed to perform better in other dimensions. However, there was no significant difference in engagement across the three English proficiency groups. One possible explanation might be that behavioral engagement in online EFL teaching might be influenced by numerous external factors, such as comparatively weaker supervision mechanism than face-to-face classroom teaching, instability of network and equipment, low efficient interaction, delayed online instruction, etc. (Chapelle, 2019; Du and Qian, 2022). It is also worth considering, as some research indicates, that a lower level of students' engagement was an inevitable problem during the COVID-19 pandemic (Yang et al., 2020) since perceived COVID-19 event strength and perceived stress can negatively influence learning engagement (Zhao et al., 2021). Nevertheless, this survey did not observe such impacts caused by external events and internal pressures, which needs to be further explored.

This survey also uncovered that motivation, engagement, strategy, and deep learning did differ between students in BE and students in PCE groups. One of the main possible reasons might be distinct curricula based on different talent training programs. BE is a core course for students who majored in TCFL, focusing on increasing mastery of language knowledge, language application skills, and cross-cultural competence, whereas PCE is a public general English course for non-English major students aiming to teach vocabulary, grammar, text, etc., and develop basic language skills. In this vein, students in BE might pay more attention and effort to EFL learning, which is visibly related to their future professional development, thereby contributing to a higher level of motivation, engagement, strategy, and deep learning. Similarly, students in ELS, which is designed to develop academic communication to ensure future academic success at overseas universities, also performed better than the PCE group in engagement. In addition, although students in PCE struggled with passing CET-4 and CET-6, in the short term, a relatively less strong correlation with their majors might undermine their efforts and attention in EFL learning, resulting in low motivation, engagement, strategy, and deep learning. Interestingly, there was no difference in competence across the three groups, even if the average rate of passing CET-4 in BE groups is higher than in others. This might be due to the underestimated self-evaluation as we discussed above.

Since some literature has demonstrated that visions are instrumental in driving human behavior in the present (Markus and Nurius, 1987), this study considered vision as one of the variable to observe its impact on deep learning in online EFL teaching. The vision here was initially set as three types of dreamed future states after graduation: hunting jobs, further studying, and self-employment, which imply different requirements for English proficiency. Results suggest that students in FS had a higher level of motivation than other groups, suggesting that students having a vision of being more proficient English speakers (i.e., FS group) presented stronger motivation to learn. This finding partly supported the motivational self system (L2MSS) theory advocated by Dörnyei (2009), highlighting the motivational potential of vision in the field of SLA. Moreover, it was discovered that students with a vision of being more proficient in English had a higher level of strategy, competence, and deep learning, which indicate that a strong vision might galvanize them to conduct deep learning strategy. Consequently, advanced competencies became much easier to acquire, and ultimately students were more inclined to access deep learning. This further confirmed the capacity of a vision of an ideal L2 self to motivate learning (Henry, 2011; You and Dörnyei, 2016). However, no significant differences in engagement existed across the three groups, similar to the results in English proficiency groups, which have been discussed above.

### Promoting deep learning in the online EFL teaching context

Based on findings in this survey, referring to the four-dimension theoretical model of deep learning in online EFL teaching, this study proposed five proper instructional strategies to promote students' deep learning. First, teachers should give top priority to cultivating students' directional competence of deep learning by organizing creative multi-facet online tasks and activities incorporating four main language application skills, or through conducting situational teaching approach and project-based approach to promote internalization and application of language knowledge, or by designing online learning navigation to gradually guide students' thinking, perceiving and behaving toward deep learning goals. Additionally, teachers can increase the number of open-ended questions in tests to develop students' English high order thinking and critical thinking. Second, teachers should take efficient measures to improve students' engagement in three stages of online EFL teaching. Specifically, teachers can make full use of ICT-assisted teaching platforms in China context or LMSs (e.g., Blackboard) to manage and supervise class by randomly taking online attendance, asking questions, interacting, giving tests, etc. Teachers can also establish a highly interactive and harmonious learning community with more teachers' emotional support to meet students' learning needs and promoting investment (Liu et al., 2021). Additionally, during the COVID-19 pandemic, strengthening students' growth mindset and reducing students' perceived stress can promote students' engagement (Zhao et al., 2021). Third, teachers should make efforts to help students exploit deep LLS, especially skill-based cognitive characters, which may be realized during implicit or explicit instruction in online EFL teaching, for example, choreographing online learning tasks and projects (e.g., self-reflection, KWL chart) to provide abundant opportunities for students to practice and evaluate strategies (Gerami and Baighlou, 2011). According to the comparative analysis, less proficient students, students in PCE course, and students with less strong English-related vision need more attention. Moreover, attempts to maintain an all-English environment in online or offline classes can also contribute to practicing skill-based strategies. Fourth, teachers need to create and maintain students' diverse language learning motives directed to deep learning through providing high-quality online materials and resources about English society, culture, customs, etc., to stimulate students' integrative motives. Teachers can also increase students' expectancy of success and goal-orientedness (Dörnyei, 2001) or utilize ICT to build and enhance vision to energize and maintain deep language learning (Adolphs et al., 2018). Fifth, since deep learning may differ across variables, teachers should exploit ICT and big data as well as LMSs (e.g., Blackboard) to manage, track, and record students' learning behaviors and analyze their various learning preferences, aiming to conduct individualized online EFL teaching to promote students' deep learning effectively.

###  Limitations and future research

It is significant to recognize potential limitations of the study, which may hopefully amount to directions for future research. First, although the correlated four-dimension theoretical model of deep learning was supported by ample literature and confirmed through EFA and CFA, suggesting the fit of the model was adequate, we still hope that the fit of the model can be further replicated in larger diverse sample sizes to establish the generalizability for different countries/regions or in various contexts of EFL teaching focusing on specific skills (listening, reading, etc.). Longitudinal research is also needed to validate the model in the future. Second, theoretic junctions of deep learning dimensions (e.g., the motivation, the strategy) and related cutting-edge SLA theories may become promising research directions to further explore, to illustrate, from a complex dynamic perspective, whether or how the motivation of deep learning is related to language learners' motivational directed currents (DMCs) (Muir and Dörnyei, 2013). Third, regarding the survey, the self-reported measurement might result in overrating or underrating real status, therefore, classroom observations and interviews can be adopted to collect more qualitative data to comprehensively investigate deep learning and dig into more in-depth reasons behind survey results. Fourth, unfortunately, the COVID-19 pandemic limited the scope of investigation in this study, which needs to be expanded in the future relevant surveys.

## Conclusion

This study exerted pioneering efforts to understand, investigate, and promote deep learning in the online EFL teaching context. The present study proposed the four-dimension model hypothesis of deep learning involving the motivation of deep learning, the engagement of deep learning, the strategy of deep learning, and the directional competence of deep learning, which was empirically validated as a positively correlated model with high composite reliability and good convergent validity by using EFA and CFA. An additional survey reported that the current level of college students' deep learning in online EFL teaching context reached median value, with the lowest mean of directional competence and the highest mean of motivation; students presented more instructional motives, neglect of deploying skilled-based cognitive strategies, and deficiency of language application skills and problem-solving skills; there existed some statistically salient differences in deep learning level and other four sub-dimensions across grades, English proficiency, EFL course, and vision groups; students presented relatively low engagement regardless of distinct English proficiency and visions and similarly low directional competence across grade and EFL course groups. In the end, this study attempted to explicate the main results and proffered five promotion instructional strategies to boost students' deep learning in the online EFL teaching context.

Overall, this study tries to clear away theoretical muddle in deep learning construct in language education and prove the latent rationality of the converge of deep learning concept and SLA theories, hopefully, proffering new insights into the theoretical and empirical development of deep learning in language education. The survey findings and instructional strategies may be useful for EFL teachers to implement efficient and individualized online EFL teaching to boost students' deep learning and further remedy problems of low effectiveness in ICL-assisted EFL teaching. The standardized instrument developed in the present study may also serve as a valuable tool for other researchers interested in this domain.

## Data availability statement

The raw data supporting the conclusions of this article will be made available by the authors, without undue reservation.

## Ethics statement

The studies involving human participants were reviewed and approved by College of Humanities and Arts, Tianjin University of Finance and Economics Pearl River College. The patients/participants provided their written informed consent to participate in this study. Written informed consent was obtained from the individual(s) for the publication of any potentially identifiable images or data included in this article.

## Author contributions

RJ was fully in charge of reviewing literature, conceiving the study, conducting a questionnaire survey, analyzing statistics, writing and revising the manuscript, etc.

## Funding

This work was supported by the Major Program of Tianjin University of Finance and Economics Pearl River College under Grant No. ZJZD21-07.

## Conflict of interest

The author declares that the research was conducted in the absence of any commercial or financial relationships that could be construed as a potential conflict of interest.

## Publisher's note

All claims expressed in this article are solely those of the authors and do not necessarily represent those of their affiliated organizations, or those of the publisher, the editors and the reviewers. Any product that may be evaluated in this article, or claim that may be made by its manufacturer, is not guaranteed or endorsed by the publisher.
