# Supplementary material for: Understanding, Investigating, and promoting deep learning in language education: A survey on chinese college students' deep learning in the online EFL teaching context
Source: Front Psychol. 2022 Sep 7;13:955565. doi: 10.3389/fpsyg.2022.955565 (PMC9490376; doi:10.3389/fpsyg.2022.955565)
Supplement: Supplementary file 1 [file Data_Sheet_1.PDF]

APPENDIX. DESCRIPTIVE STATISTICS OF OVERALL 22 ITEMS

Variable	N	M	SD
C2. I think I acquire English application skills (i.e., listening, speaking, reading, translating , etc.)	474	2.7975	.83850
C4. I think I have English critical thinking(i.e., I can think, analyze , distinguish, persuade, and explain, etc., information and ideas in English)	474	2.8882	.88573
C5. I think have the ability to acquire, filter and extract information online in English	474	3.1287	.82911
C3. I think I have English learning autonomy (i.e., I can regulate my English learning independently)	474	3.1709	.81627
C6. I think I can solve practical problems in English in specific context	474	2.7068	.87270
C1. I think I have mastery of basic English language knowledge (e.g., grammar, vocabulary , etc.)	474	2.8101	.85887
S11. I actively pose related questions on what I have learnt for analysis.	474	3.1582	.81104
S3. I regularly reflect on my English learning to avoid making similar mistakes.	474	3.3608	.84196
S12. I challenge my opinion on the prior questions and knowledge.	474	3.3186	.83387
S10. I try to use my English in real situation, such as write emails, send messages, chat with native speakers, etc.	474	2.8165	.87608
S2. I look for words in my own language that are similar to new words in English	474	3.3544	.83837
S9. I participate in group discussions with students to better understand learning content from different perspectives.	474	3.3249	.80952
E5. In online class, I ask question or contributed to course discussion or project in other way	474	3.1751	.83586
E6. In online class, I actively answer questions raised by teachers.	474	2.9852	.90441
E4. Before online class, I preview what I will learn and main active mood.	474	3.2637	.85779
E7. After online class, I discuss unsolved questions or other English learning content with teachers or my classmates.	474	2.9895	.85398
E2. After online class, I review and summarize key ideas or concepts.	474	3.1097	.86057
M2. I learn English diligently in order to pass standardized English tests (e.g., CET4, CET6, TOEFL, IELTS, etc.)	474	3.9515	.79624
M3. I learn English diligently to promote personal development and improve competiveness in the future.	474	4.2743	.85075
M5. I think it's important and meaningful to speaking English fluently.	474	3.9030	.78508
M1. I think it's important to learn English in order to know more about culture and arts of its speakers	474	3.5865	.75125
M4. I feel happy and satisfied when I complete difficult and challenging English learning task or project.	474	3.9051	.80528
